# Clinical implementation and performance evaluation of a motorized MR‐compatible OCTAVIUS 4D system for SBRT patient‐specific QA on a 1.5 T MR‐LINAC

**DOI:** 10.1002/acm2.70736

**Published:** 2026-08-10

**Authors:** Clement Chevillard, Marie Blanchet, Cyrielle Mella, Robin Sauvinet, Rezart Belshi, Ivan Taranenko

**Affiliations:** ^1^ Department of Radiation Oncology Service of Medical Physics, Institut Curie Saint Cloud France; ^2^ Elekta Limited, Cornerstone Crawley West Sussex UK

**Keywords:** MR‐LINAC, OCTAVIUS 4D phantom, OCTAVIUS detector 1500 MR, patient‐specific quality assurance

## Abstract

**Background:**

Magnetic resonance–guided radiotherapy using 1.5 T MR‐LINAC systems introduces specific constraints for patient‐specific quality assurance (PSQA), requiring MR‐compatible dosimetric devices capable of accurate three‐dimensional dose verification. While previous studies have characterized the dosimetric performance of MR‐compatible detector arrays, the clinical implementation of rotational three‐dimensional QA systems in MR‐guided workflows remains insufficiently documented.

**Purpose:**

This study investigated the clinical implementation and practical performance of a motorized MR‐conditional OCTAVIUS 4D system for stereotactic body radiotherapy (SBRT) PSQA on a 1.5 T MR‐LINAC.

**Methods:**

The MR‐conditional OCTAVIUS 4D phantom equipped with a 1500 MR ionization chamber array was implemented for PSQA measurements on a Unity 1.5 T MR‐LINAC (Elekta, Stockholm, Sweden). A total of 40 clinical step‐and‐shoot treatment plans were analyzed with an SBRT prescription. The system was evaluated in terms of three‐dimensional dose reconstruction accuracy under clinically relevant off‐axis condition, angular positioning consistency with the gantry positioning, and integration into clinical workflow. Measured dose distributions were compared with treatment planning system (TPS) calculations using a 3D gamma analysis with a local 2.5%/2.5 mm criterion and a 10% dose threshold. Independent angular measurements were additionally performed using a digital inclinometer.

**Results:**

The system demonstrated high agreement between measured and calculated dose distributions, with a mean gamma passing rate of 98.3% (local 2.5%/2.5 mm). All plans met institutional acceptance criteria (GPR ≥ 95%). Independent angular measurements demonstrated excellent agreement between gantry angle, phantom angle, and software‐reported angle, with deviations below 0.6° and no systematic bias observed. No visible dependence of gamma passing rate on radial off‐axis target positioning was observed within the investigated clinical range. The evaluated beam arrangements included irregular angular spacing and clockwise/counterclockwise gantry transitions during delivery, supporting effective synchronization between the OCTAVIUS 4D MR system and the Unity gantry under clinical step‐and‐shoot conditions.

**Conclusions:**

The motorized MR‐conditional OCTAVIUS 4D system demonstrated clinically feasible implementation for rotational three‐dimensional PSQA for SBRT on a 1.5 T MR‐LINAC. The system provided consistent dose reconstruction accuracy and reliable angular positioning under clinically relevant delivery conditions, supporting its integration into routine MR‐guided radiotherapy QA workflows.

## 1 INTRODUCTION

Modern radiotherapy treatments routinely use intensity‐modulated techniques, such as volumetric modulated arc therapy (VMAT) and intensity‐modulated radiotherapy (IMRT), as the standard for clinical practice. These techniques enable high target conformity, steep dose gradients, and improved sparing of organs at risk (OARs). Such dosimetric advantages arise from the complex and dynamic nature of treatment delivery, including multileaf collimator (MLC) motion, dose‐rate modulation, and for VMAT, variations in gantry positioning speed. However, the increased delivery complexity renders intensity‐modulated treatments more sensitive to mechanical uncertainties, dose calculation errors, and delivery inaccuracies. Consequently, independent verification of the planned dose delivery is required to ensure patient safety. The implementation of a Patient‐Specific Quality Assurance (PSQA) program is therefore considered essential prior to clinical application, as recommended by international guidelines, including AAPM Task Group reports TG‐119 and TG‐218 and publication from the International Atomic Energy Agency (IAEA).^1^, ^2^, ^3^, ^4^ PSQA aims to compare the dose calculated by the treatment planning system (TPS) with the dose delivered by the treatment machine. A variety of verification systems are employed, including but not limited to electronic portal imaging devices (EPID), 2D array matrices (with diode detectors or ion chambers), 3D phantoms (Delta4 (ScandiDos) or ArcCheck (Sun Nuclear, Melbourne, USA)), and hybrid detectors (Octavius 4D (PTW, Freiburg, Germany)) capable of reconstructing the measured dose in 3D.^5^, ^6^

Emerging technologies such as MR‐guided linear accelerators (MR‐LINAC) integrate high‐resolution magnetic resonance imaging with a clinical linear accelerator to enable real‐time soft‐tissue visualization, continuous monitoring of tumor motion, and truly adaptive radiotherapy delivery, thereby enhancing treatment precision and personalization compared with conventional image‐guided radiotherapy. In clinical practice, two major MR‐LINAC systems are commercially available: the MRIdian® LINAC (ViewRay Inc, Cleveland, OH) and the Unity® system (Elekta, Stockholm, Sweden), which differ in their magnetic field strengths, geometries, and imaging configurations but both allow MR‐based tracking and online adaptation of treatment plans.^7^, ^8^, ^9^ However, the presence of a static magnetic field imposes significant constraints on quality assurance (QA) practices. Conventional QA devices designed for standard LINACs often contain ferromagnetic components or electronics that behave unpredictably in an MR environment and therefore cannot be safely operated within or near the MR bore unless they are certified as MR‐safe or MR‐conditional^10^ for the specific field strength of the system in use.^11^ To address these challenges, several PSQA solutions have been developed in MR‐LINAC environments, including EPID based approaches,^12^, ^13^ detector arrays, and 3D or quasi‐3D measurement devices. Studies have shown, for example, that MR‐compatible detector arrays can achieve high gamma passing rates (GPR) and robust performance for PSQA in low‐field MR‐LINAC environments when carefully selected and calibrated.^14^ In addition, detector matrix–based systems have gained increasing interest due to their ability to provide efficient, reproducible, and high‐resolution dose measurements, while remaining compatible with the constraints imposed by the magnetic field. The OCTAVIUS platform, and in particular the OCTAVIUS 4D concept, a combination of a motorized rotational 3D phantom and a 2D matrix‐based detector, belongs to this category and offers the additional capability of reconstructing three‐dimensional dose distributions from two‐dimensional measurements.

Recently, PTW (Freiburg, Germany) released a new version of the motorized modular OCTAVIUS 4D phantom specially designed for rotational delivery techniques and compatible with MR‐LINACs. A recent study by Gorobets et al.^15^ evaluated the performance of MR‐compatible OCTAVIUS 2D arrays (1500 MR and 1600 MR) and concluded that both arrays demonstrated acceptable dosimetric accuracy across a range of fundamental tests. Specifically, the study reported excellent short‐term reproducibility (< 0.2 %), strong dose linearity (< 1 %), minimal dose per pulse dependency (< 0.4 %), and low monitor unit rate dependency (< 0.8 %) for the 1500 MR array, with similar performance observed for the 1600 MR array. Field size dependence was also shown to be within clinically acceptable limits (< 0.7 % for field sizes larger than 5 × 5 cm^2^) and angular response deviations remained small (standard deviation < 0.5 %), indicating consistent detector behavior across beam orientations. When subjected to clinical treatment plans, the 1500 MR achieved high GPRs consistent with standard QA criteria (≥95 % for global 3 %/3 mm and ≥90 % for global 2 %/2 mm), and demonstrated sensitivity to setup and MU errors introduced during testing.^15^ However, while prior work primarily focused on intrinsic detector array performance, the practical clinical implementation of rotational three‐dimensional QA system in MR‐LINAC environments remains insufficiently described. In particular, limited data are available regarding (i) angular positioning consistency between the rotation unit and the Unity® gantry, (ii) the impact of geometrical offset correction on three‐dimensional dose reconstruction accuracy during high‐gradient SBRT delivery with off axis measurement from the isocenter, and (iii) practical workflow integration within a 1.5 T MR‐guided radiotherapy environment.

The aim of this study was therefore to investigate the clinical implementation and practical performance of the motorized MR‐conditional OCTAVIUS 4D system combined with the 1500 MR ionization chamber array for SBRT patient‐specific QA on a 1.5 T MR‐LINAC. Emphasis was placed on workflow integration, angular positioning evaluation, and three‐dimensional dose reconstruction under clinical delivery conditions.

## 2 MATERIALS AND METHODS

### 2.1 Material

This study was conducted using an Elekta Unity MR‐LINAC (Elekta, Crawley, UK) which integrates a 7 MV flattening filter free (FFF) photon beam with a 1.5 T MR‐scanner (Philips Medical Systems, The Netherlands). The system isocenter is positioned at 143.5 cm from the radiation source with the isocenter positioned 14 cm above patient positioning device. The LINAC is equipped with a modified Agility® MLC for beam shaping with a nominal size of 0.7175 cm at isocenter, providing field sizes ranging from 0.8 cm × 0.5 cm to 57.4 cm × 22 cm at isocenter.^16^ MLC is positioned in the in‐plane direction. The Unity system delivers treatments using a step‐and‐shoot technique with discrete gantry repositioning between beam angles. The nominal maximum gantry positioning speed is approximately 6 rpm (∼36°/s). An EPID is available for QA purposes and is positioned at 265.3 cm from the source and allows a 22 cm x 9.5 cm imaging. Patient positioning device is only capable of longitudinal movement.

**FIGURE 1:**
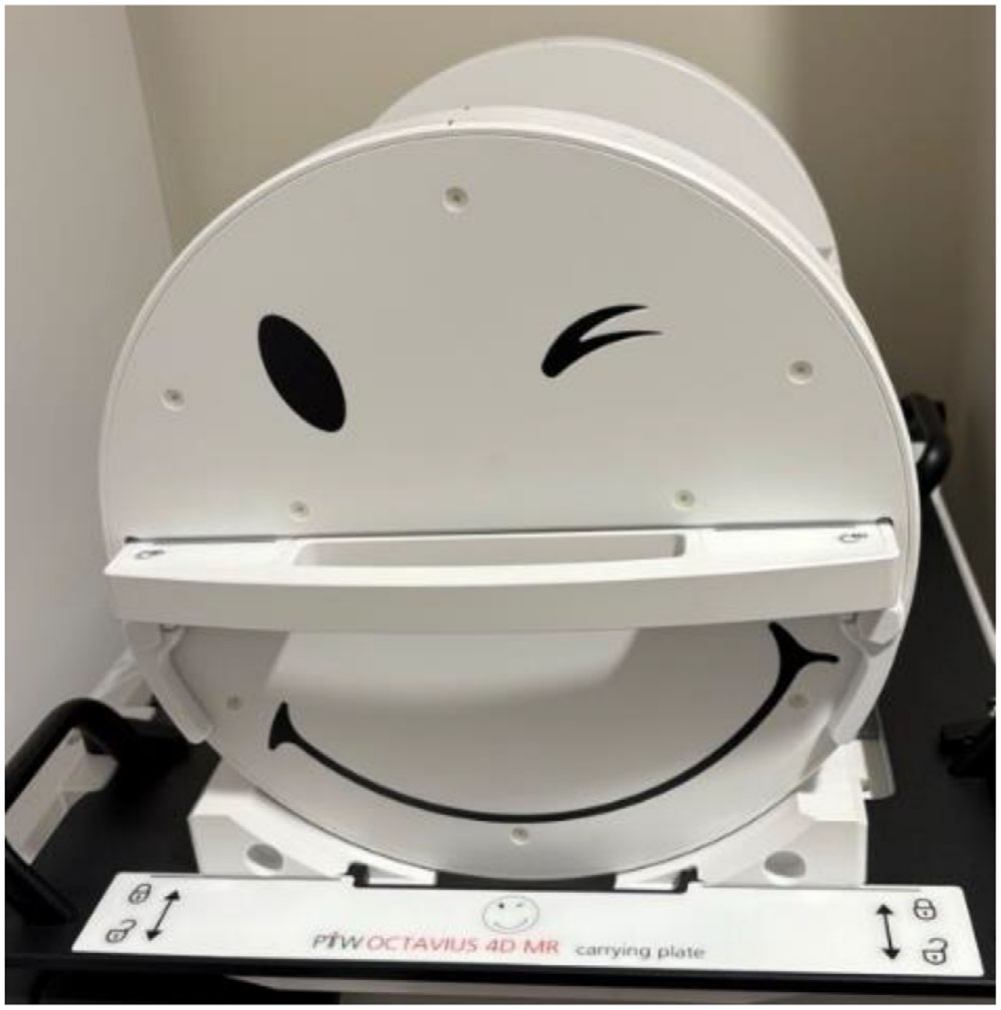
Motorized Octavius 4D MR phantom.

**FIGURE 2:**
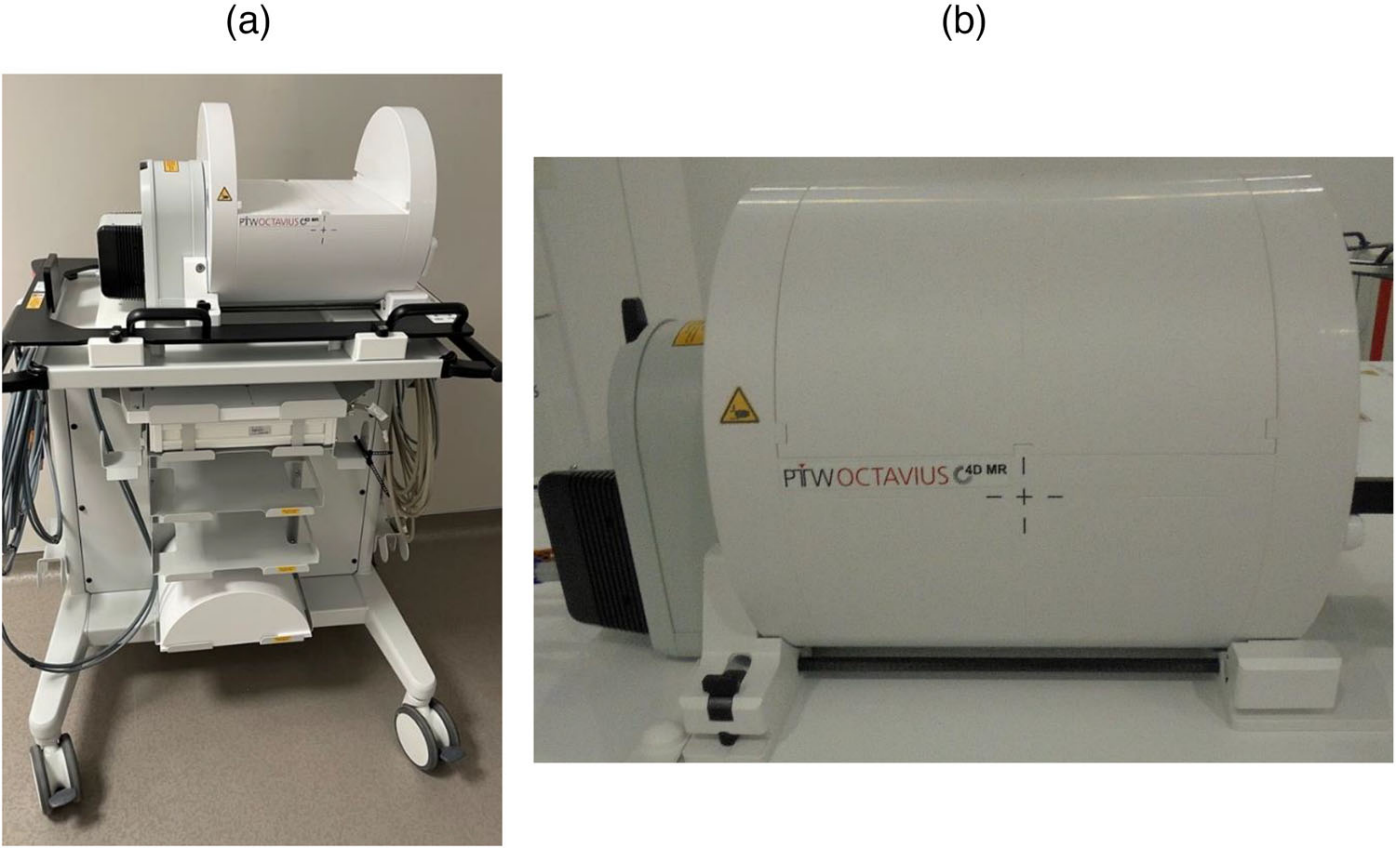
(a) Octavius 4D rotation unit stored on the trolley; (b) Octavius 4D rotation unit positionned on the UNITY treatment couch.

The new motorized OCTAVIUS 4D MR is a 3D modular phantom composed of water‐equivalent plastic (1.05 g/cm^3^) with a rotational speed up to 28°/s according to manufacturer specifications. The phantom has a central insert dedicated to a detector board presented on Figure 1. In this study, the OCTAVIUS detector 1500 MR array (OD 1500 MR) was inserted into the modular phantom with the OCTAVIUS standard top (272 mm × 317 mm × 137.4 mm), corresponding to a 320 mm phantom diameter. The phantom's rotational position is synchronized with the LINAC using a network cable and a third‐party software (AngleSelectionTool V1.0.0, PTW‐Freiburg). The phantom's angular position is controlled via three different ways: manual adjustment, loading the RT plan or interfacing directly with the LINAC network (iCom mode). The phantom and the store trolley are shown in Figure 2.

The OD 1500 MR array consists of 1405 ionization chambers arranging a 27 cm × 27 cm field size. Each detector has a size of 4.4 mm × 4.4 mm × 3 mm with a 0.06 cm^3^ detector volume. Detector spacing is 7.1 mm center‐to‐center. A calibration procedure for absolute dose is performed by PTW using a ^60^Co beam. The calibration file provided by PTW is used to correct ionization chamber response over the array and to convert ionization into dose.

### 2.2 QA plan preparation

**FIGURE 3:**
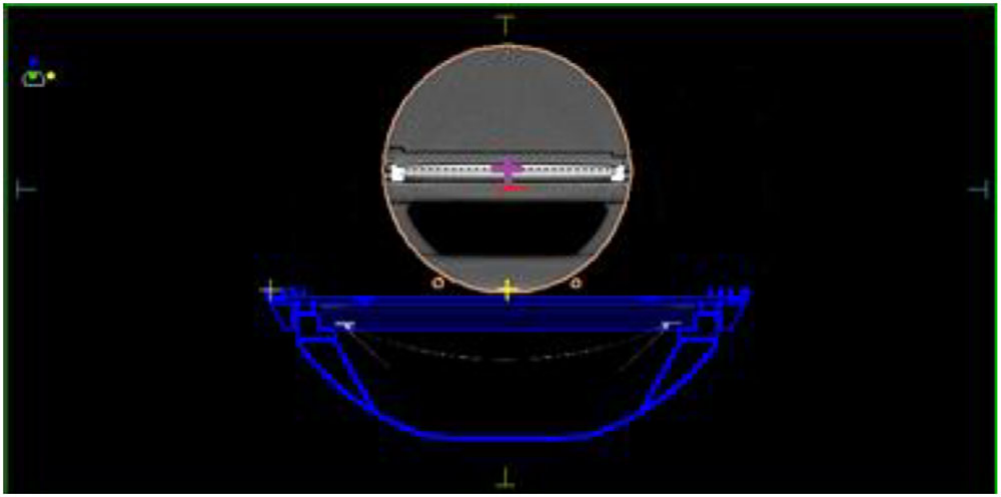
A slice of the CT image showing the Octavius 4D phantom, the array, external contour and treatment couch structures imported in MONACO.

The OCTAVIUS 4D rotation unit and the OD 1500 MR were scanned with a 2‐mm slice thickness using the radiotherapy CT simulator (Brilliance CT Big Bore, Philips Healthcare, Andover, Massachusetts). The phantom and the array in the allocated position were placed on the UNITY treatment couch positioned on top of the CT simulator couch. The scan was then imported in MONACO TPS version 6.2. External contouring was performed using a threshold method. An electron density value of 1.05 g/cm^3^ was assigned to the structure as recommended by the manufacturer. Couch structures were imported without the mattress as conventionally used with patient workflow. The center of the phantom was defined in MONACO to facilitate beam alignment during QA process as shown by the purple arrow on Figure 3. All beams were centered to this point during QA process. Figure 3 shows a slice of the CT image showing the phantom, the array, external contour and treatment couch structures in MONACO TPS.

**FIGURE 4:**
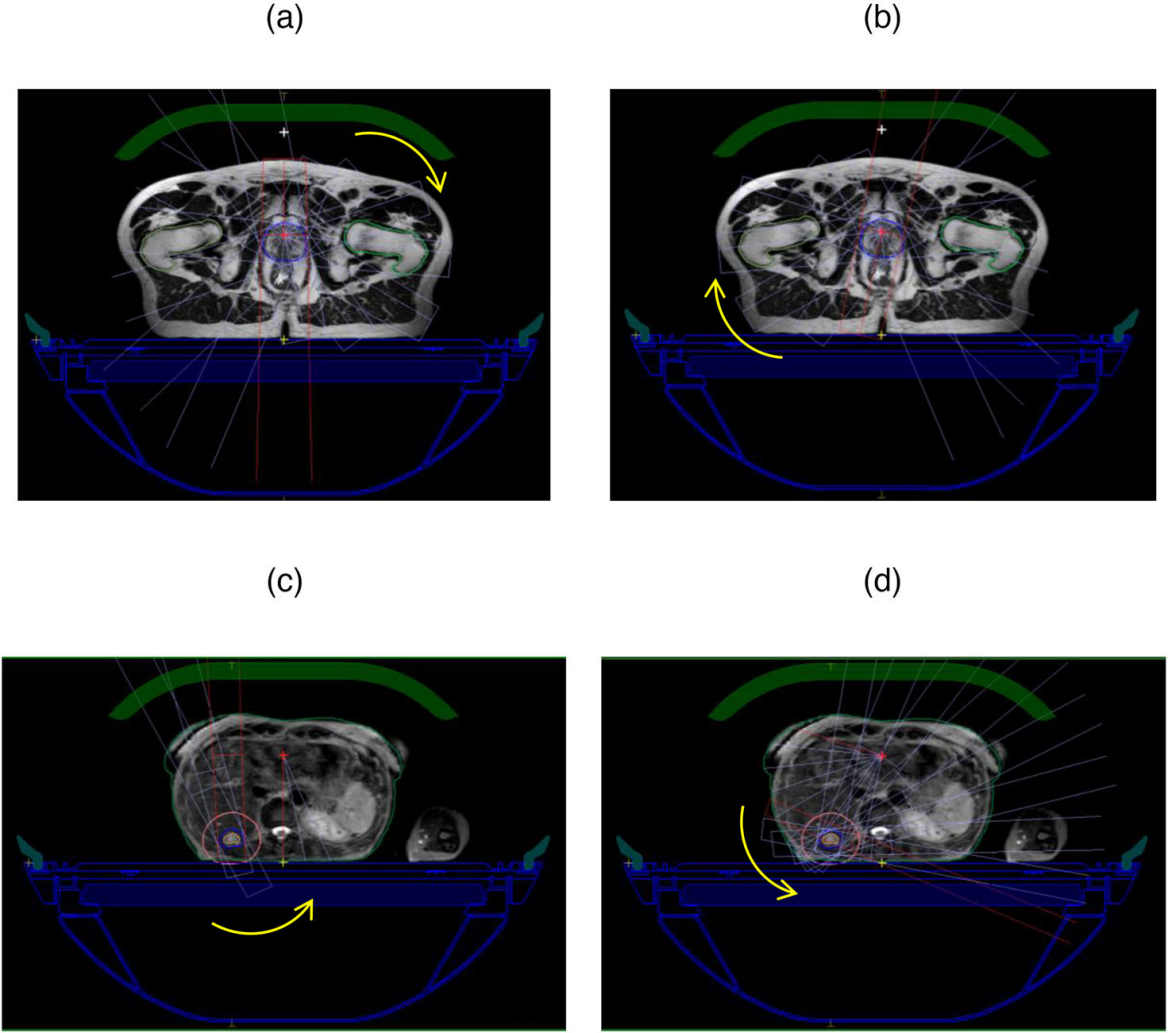
Representative beam arrangements for prostate (a, b) and a liver (c, d) SBRT plans delivered on the Unity MR‐LINAC. For visualization purposes, beam configurations are separated into two angular sequences for each treatment site. The red beam and red arrow indicate respectively the first delivered beam on the angular sequence and the isocenter, while the yellow arrows indicate the direction of gantry rotation during delivery.

**FIGURE 5:**
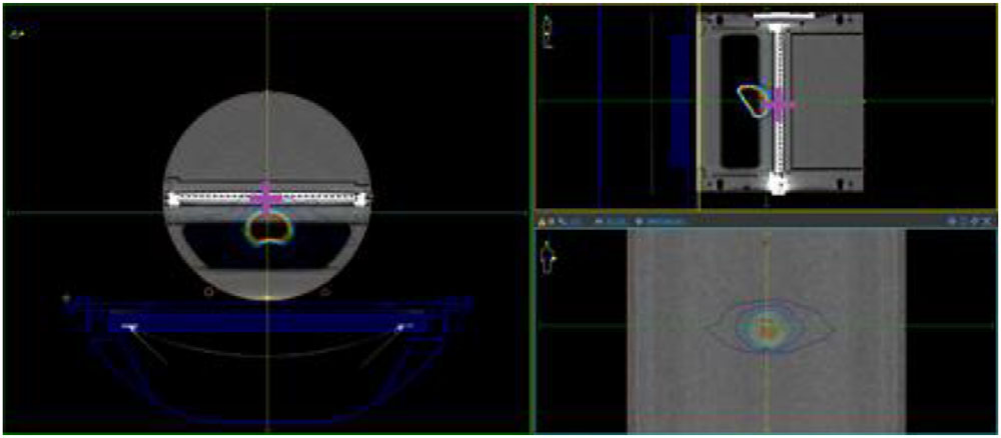
QA plan calculated on the Octavius 4D MR phantom for a prostate plan. Purple arrow mark the center of the phantom.

40 IMRT plans using step‐and‐shoot technique for various stereotactic body radiation therapy (SBRT) treatment (33 prostates, 2 pancreas and 5 livers) were selected for this study. Prescription and margins for the planning target volume are reported in Table 1. The treatment plans had 11 to 15 beams depending on the target location. Prostate SBRT plans typically involved approximately regular angular spacing by approximately 24° with clockwise/counterclockwise direction changes during delivery, whereas liver SBRT plans included partial‐arc‐like beam distributions with smaller angular separations as small as 10°‐12° between successive beams. For each evaluated plan, a radial off‐axis distance was calculated as the Euclidean distance between the PTV center and the plane isocenter, using the lateral and vertical displacement components. Representative beam arrangements and examples of off‐axis target positioning are illustrated in Figure 4 with the red arrow representing plan isocenter and PTV is shown in blue. Number of segments per beam were between 1 and 10 depending on the complexity of the treatment plan. Segment widths were kept at a minimum of 0.75 cm and the number of monitor units (MU) are set at a minimum of 8MU per segment. Total of MU per plan were between 1200 and 2900 MU. Plans were calculated using a 3 mm dose grid in all directions, a Monte Carlo (MC) dose engine with a statistical uncertainty (SU) of 1% per plan with the patient lookup table. For all the evaluated plan, a QA plans was calculated in the TPS with a dose grid of 3 mm, and a MC dose engine with 0.3% SU per plan and a phantom lookup table^14^ instead of patient lookup table usually selected for patient clinical plans. Patient and phantom lookup table differ by the material composition employed for mass attenuation coefficient used with MC calculation. The plan isocenter position is fixed with the Unity system (14 cm above the treatment couch) then the QA plan isocenter is centered in longitudinal direction of the array. Due to the fixed treatment isocenter geometry of the Unity MR‐LINAC, some QA plans involved asymmetric target positioning relative to the detector plane, resulting in varying clinically relevant off‐axis measurement conditions. DICOM RT plan and total dose files were exported in order to compare to the delivery dose. Figure 5 illustrates the dose calculated on the Octavius 4D phantom for a prostate QA plan. The purple arrow marks the center of the phantom.

**TABLE 1:** Prescription, margins and PTV volume for various tumor site treated at the UNITY.

Tumor location	Prescription [(Gy/fraction) × number of fraction]	Margins (CTV to PTV) [mm]	Mean PTV volume [cc]
Prostate (Patient 1–33)	7.25 × 5	3	108.8 ± 49.1
Pancreas (Patient 34–35)	7 × 5	3	17.66 ± 8.3
Liver (Patient 36–39)	10 × 5	3 to 5	14.4 ± 4.94
Liver (Patient 40)			158

### 2.3 QA plan measurement

**FIGURE 6:**
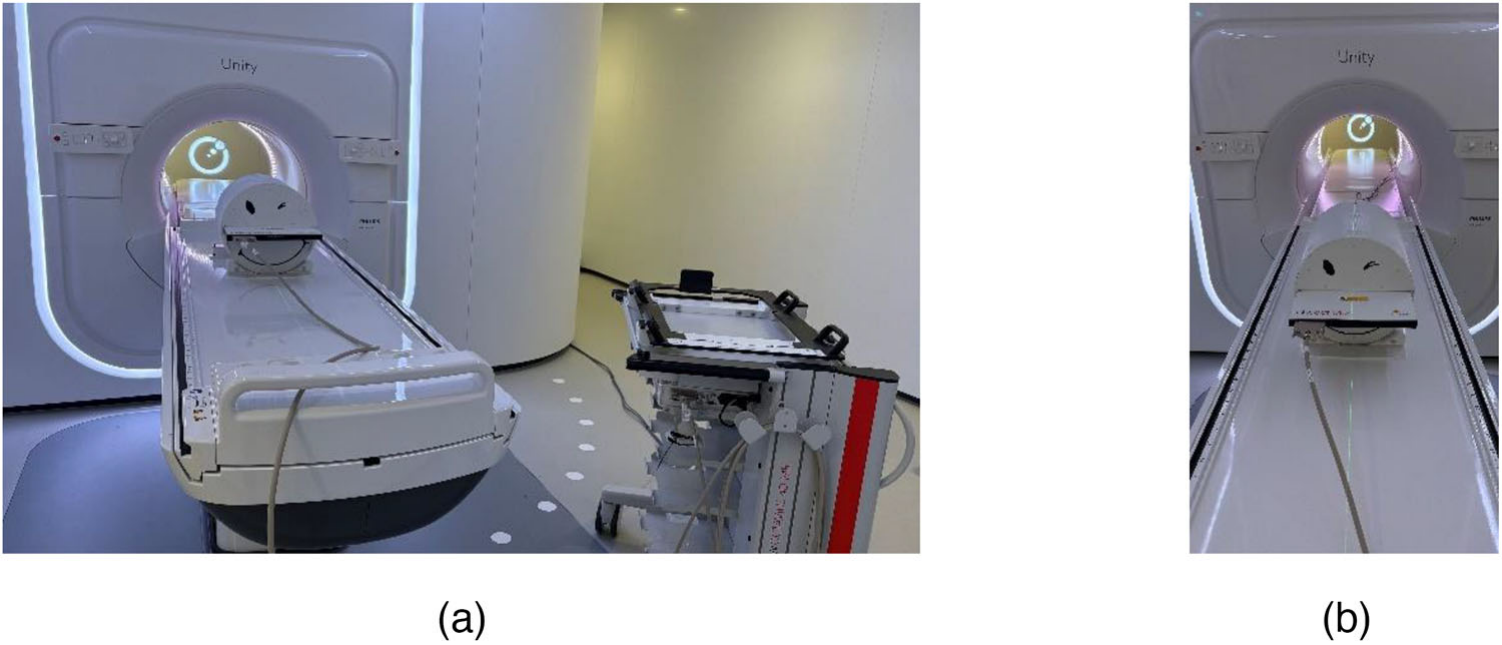
(a)OCTAVIUS 4D rotation unit and the trolley; (b) OCTAVIUS 1500 MR array inside the rotation unit.

The phantom was positioned on the treatment couch with the detector array positioned 22 mm above the isocenter of the UNITY system. Figure 6 shows the setup of the OCTAVIUS 4D rotation unit, the trolley storing the control unit and the array interface and the array inside the rotation unit.

**FIGURE 7:**
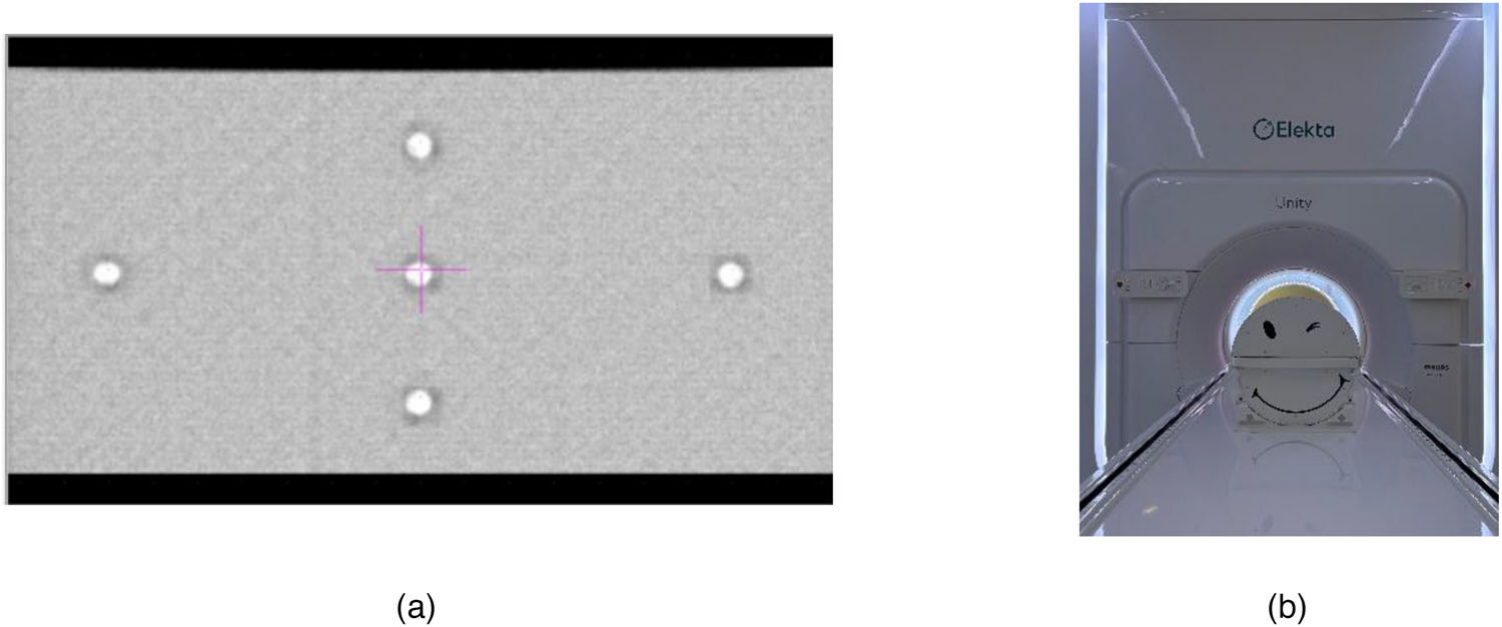
(a) MV image of the marker plate; (b)marker plate positioned inside the phantom.

To align the phantom in the plan isocenter of LINAC a marker plate is provided and the MV panel is used to position the phantom at isocenter. Figure 7 shows the MV image of the marker plate centered at isocenter marks by the purple arrow (Figure 7a) and the phantom positioner on the treatment couch (Figure 7b). Phantom positioning was considered acceptable when the marker plate was aligned within approximately 1 mm of the Unity isocenter on MV imaging.

Prior to any measurement, a 25 cm × 25 cm field size with 500 MU were used to warm up the array. Measurements were conducted using PTW Verisoft V8.1.1 (PTW‐Freiburg, Germany). The array detector was cross‐calibrated by delivering a 10 cm × 10 cm field with a prescribed dose of 2 Gy at the array calculated in the TPS with a SU of 0.3% per control point with the phantom lookup table. The corresponding MU were then delivered to the array and the expected dose was provided to Verisoft to perform the cross‐calibration factor. All measurements were acquired for the entire plan (all beams delivered to the array). Prior to any patient QA plan acquisition, a 10 cm × 10 cm field size with 200 MU were acquired to evaluate the percentage depth dose (PDD)) reconstruction in the phantom against the dose from the TPS in order to verify the correct relative electron density used for dose calculation and to minimize deviation from the expected value.

To assess the angular synchronization between the OCTAVIUS 4D system and the MR‐LINAC gantry, independent measurements were performed using a digital inclinometer placed on the surface of the phantom. Measurements were acquired at predefined gantry angles (0°, 45°, 90°, 135°, 180°, 210°, 270°, and 310°). Due to the orientation of the inclinometer, measured angles were referenced to the horizontal plane and converted to gantry angles using a geometric transformation depending on the angular quadrant.

### 2.4 QA plan analysis

Verisoft software is able to compile dose from multiple measurements and recalculate dose in 3D for coplanar and noncoplanar geometries as well as off‐axis conditions allowing verification of plan modulation consistency throughout rotation. For dose reconstruction, the Verisoft geometrical offset correction option was activated. This option compensates for the geometrical displacement between the detector measurement plane and the treatment isocenter during phantom rotation. During rotational delivery, the software applies a coordinate transformation to reconstruct the measured dose at the effective detector position relative to the radiation isocenter. In the present configuration, the detector plane was positioned 22 mm above the treatment isocenter during measurements. The offset correction therefore allows the reconstructed three‐dimensional dose distribution to be calculated at the effective detector plane position relative to the treatment isocenter. Dose reconstruction is based on PDD data for field size ranging from 2 cm × 2 to 26 cm × 26 cm or the largest possible square field size. In this study, the default PDD for a 7 FFF beam energy provided by PTW with the software was used. To evaluate the dosimetric agreement between the measured and calculated dose a 3D γ evaluation method implemented in Verisoft was used with a local γ criterion of 2.5%/2.5 mm and cut‐off values of 10% of the maximum dose to the entire volume and with the “2^nd^ and 3^rd^ pass” option activated^17^ to avoid false positive results caused by the resolution of the detector and a gamma passing rate (GPR) set at 95%. The AAPM recommends, for IMRT PSQA, a dose threshold of ≥10% of the prescription dose, a dose difference (DD) of 3%, and a distance‐to‐agreement (DTA) of 2 mm.^18^ The TG‐218 criteria (3%/2 mm) remain widely adopted for IMRT PSQA and may facilitate comparison of results across institutions. However, considering high dose gradient with SBRT treatment, tighter criteria are suggested to improved sensitivity to detect errors but each institution is left to tailor criteria locally. In our clinic, we adopted for SBRT treatment a local 2.5%/2.5 mm with a 10% dose threshold with a GPR greater than 95% without the automatic alignment option used. These criteria were applied for MR‐guided workflow.

## 3 RESULTS

### 3.1 Basic validation measurements

**FIGURE 8:**
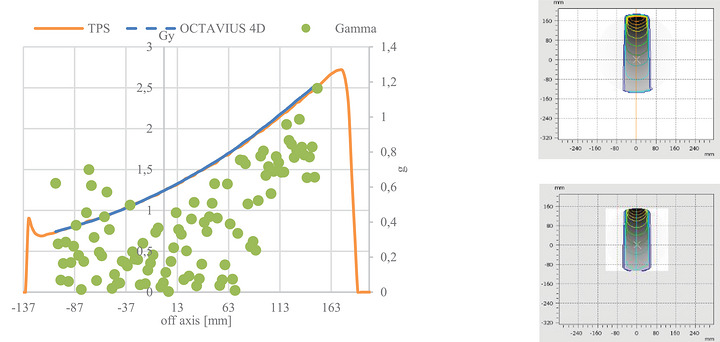
Static 10 cm × 10 cm field at gantry 0°, transverse plane: central axis depth dose distribution (left) obtained with the Octavius 4D 1500 (blue curve), compared with TPS (orange curve). GPR in the complete plane was 97.1% (1.5%/1.5 mm local).

**FIGURE 9:**
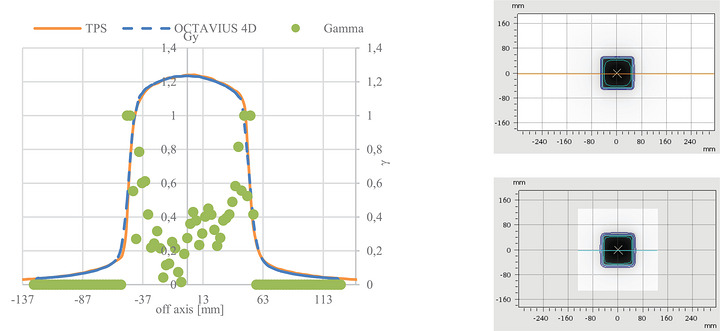
Static 10 cm × 10 cm field at gantry 0°, coronal plane: isocentric cross profile (left) obtained with the Octavius 4D 1500 MR (blue curve), compared with TPS (orange curve). GPR in the complete plane was 92.2% (1.5%/1.5 mm local).

Reconstructed profiles at plan isocenter and reconstructed depth dose profile were compared with the corresponding profiles obtained from the TPS. Measurements were repeated before each patient QA acquisition. Figure 8 and Figure 9 show the result of a static 10 cm × 10 cm beam in transversal plane and in coronal plane respectively, evaluated with Verisoft software. Good agreement was observed between the reconstructed dose and the TPS calculation with a 3D GPR of 97.1% and 92.2% respectively in transversal and coronal plane (1.5%/1.5 mm, local). The validation measurements with a static 10 cm × 10 cm field demonstrated excellent agreement between the OCTAVIUS 4D MR reconstructed dose and the TPS calculation, with mean 3D GPR of 94.0% (1.5 %/1.5 mm local). The observed dose differences at gantry 0° remained within the acceptance criteria specified by the manufacturer for reconstructed dose verification. In the example provided in Figure 8 the dose difference was 0.16%.

**FIGURE 10:**
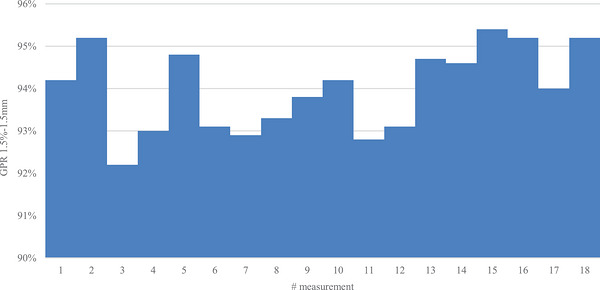
3D GPR analysis over 18 measurements with the OD 1500 MR and the OCTAVIUS 4D MR for a 10 cm × 10 cm field size at gantry 0° (1.5%/1.5 mm local).

Figure 10 shows the 3D gamma analysis of the dose matrix reconstruction for different 10 cm x 10 cm static field size at gantry 0° with the OCTAVIUS 4D and OD 1500 MR for the local 1.5%/1.5 mm with 10 % dose threshold criteria measured at different time points prior to patient‐specific QA acquisitions. The results demonstrate a very good agreement between the dose from the TPS and the reconstructed dose with an average GPR of 93.98 ± 1% [92.2%‐95.4%].

The comparison between gantry angle, OCTAVIUS‐reported angle, and independently measured angle showed excellent agreement, with a mean deviation of −0.3° and a maximum deviation below 0.6°, and no systematic bias observed (Table 2). These measurements demonstrate accurate and reproducible angular positioning of the phantom throughout the investigated angular range.

**TABLE 2:** Comparison between gantry angle, OCTAVIUS‐reported angle, and independently measured phantom angle.

Gantry angle (°)	Octavius reported angle (°)	Measured angle (°)	Δ (Gantry‐measured) (°)
0	0	0.35	+0.35
45	45	44.4	−0.6
90	90	89.45	−0.55
135	135	134.4	−0.6
180	180	179.55	−0.45
210	210	209.8	−0.2
270	270	269.65	−0.35
310	310	310	0

### 3.2 Patients data analysis

**FIGURE 11:**
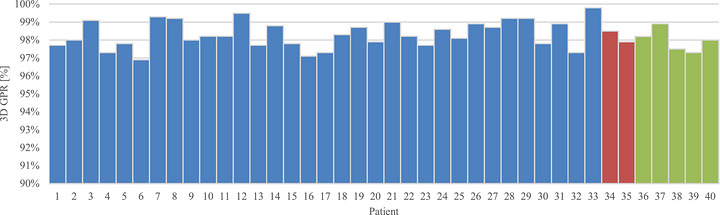
3D GPR of 40 plans measured with the Octavius 4D and the OD 1500 MR. (blue: prostate; red: pancreas; green: liver).

For the 40 clinical plans evaluated (33 prostates, 5 livers, 2 pancreas), Figure 11 shows the results of the 3D GPR of the 40 plans with an average 3D gamma pass rate of 98.3 ± 1% [96.9%–99.8%] using a 2.5 %/2.5 mm local criterion and a 10 % dose threshold. All plans satisfied the acceptance criteria of 95 %, consistent with standard clinical QA practice.

**FIGURE 12:**
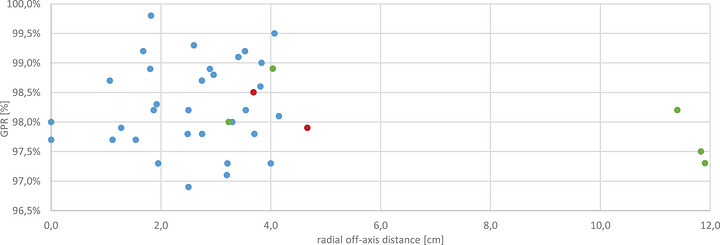
Relationship between radial off‐axis distance and 3D GPR for the 40 evaluated SBRT QA plans. (blue: prostate; red: pancreas; green: liver).

No visible dependence of gamma passing rate on off‐axis target positioning was observed within the investigated clinical range. The investigated plans included clinically relevant asymmetric target configurations, particularly for liver and pancreas SBRT cases, resulting in radial off‐axis distances up to 11.9 cm. Figure 12 shows the relationship between 3D gamma passing rate and radial off‐axis distance.

In addition, the investigated beam arrangements involved repeated gantry repositioning, irregular angular spacing, and clockwise/counterclockwise delivery transitions during step‐and‐shoot delivery. Combined with the excellent agreement observed between calculated and reconstructed dose distributions, these findings support effective synchronization between the OCTAVIUS 4D MR phantom rotation and the Unity gantry under the investigated clinical conditions.

## 4 DISCUSSION

This study evaluated the clinical implementation and performance of the motorized MR‐conditional OCTAVIUS 4D system for SBRT PSQA on a 1.5 T Unity MR‐LINAC. A key distinction between the present work and previous studies lies in the level of system integration evaluated. While prior work, including that of Gorobets et al. focused primarily on the intrinsic dosimetric performance MR‐conditional detector arrays, the current study investigated the clinical implementation of the motorized rotational QA system. This includes synchronization with MR‐LINAC gantry, the three‐dimensional dose reconstruction under SBRT deliveries with offset condition, and practical workflow in clinical implementation.

The results of the square static field size reconstructed show a very good agreement between dose calculated with MONACO and dose reconstructed for the dose measured with the array. In particular, PDD reconstructed in Verisoft shows a very good agreement with TPS dose calculation.

The mean 3D gamma pass rate for patient PSQA of 98 % (2.5%/2.5 mm local) was consistent with previously published results by Gorobets et al.,^15^ who reported pass rates between 95 % and 99 % (3%/3 mm global) for similar QA plans using both 1500 MR and 1600 MR arrays on the Unity. However, our results (98.3 % at 2.5 %/2.5 mm) are slightly higher than those reported by Gorobets et al.^15^ (95%–99%) and within the range expected for QA workflows. The use of the Verisoft geometrical offset correction option was explicitly implemented and described in the present work, whereas its use was not detailed in the publication by Gorobets et al. This correction accounts for the geometric offset between the detector rotation axis and the treatment isocenter by reconstructing the measured dose at the effective detector plane position. The evaluated clinical plans included asymmetric target configurations relative to the detector plane and to the Unity isocenter, particularly for liver and pancreas SBRT cases, resulting in clinically relevant off‐axis measurement conditions. These results confirm that the MR‐compatible design of the 1500 MR array maintains dosimetric accuracy in the presence of a strong magnetic field, in line with findings from Renkamp et al.^14^ at 0.35 T MR‐LINAC systems.

The angular positioning assessment demonstrated excellent agreement between gantry angles, OCTAVIUS‐reported angle, and independently measured phantom angle, with a maximum deviation below 0.6° and no systematic bias observed. These measurements primarily demonstrated accurate and reproducible phantom angular positioning throughout the investigated angular range. In addition, the evaluated clinical plans involved repeated gantry repositioning, irregular angular spacing, and clockwise/counterclockwise delivery transitions during step‐and‐shoot delivery. Combined with the high agreement observed between reconstructed and TPS‐calculated dose distributions, these findings support the effective synchronization of the OCTAVIUS 4D MR system with the Unity gantry under the investigated clinical conditions.

No visible dependence of gamma passing rate on beam angular configuration or off‐axis target positioning was observed within the investigated clinical range. Although the present study did not include highly modulated extreme delivery conditions, the investigated plans represented clinically relevant SBRT beam arrangements encountered in routine Unity treatments.

Compared with QA methods relying on EPID or diode, the OCTAVIUS 4D MR provides a fully MR‐conditional alternative enabling integrated QA in the clinical environment. Previous reports from Romaguera et al.^19^ have highlighted similar variability for Unity QA plans, with an expected gamma pass range of 90%–99% under 2 %/2 mm criteria.

However, further work is required to fully characterize the performance of the OCTAVIUS system for very lateral irradiation geometries (off axis position greater than 12 cm from central axis) with the phantom positioned off axis or extremely small field sizes, which may affect detector response and rotational reconstruction accuracy. Regarding the positioning of the phantom, the method with the marker plate is relatively cumbersome and could benefit from dedicated positioning accessories to improve setup reproducibility. The impact of phantom positioning errors relative to the beam central axis should be considered in future investigations. Previous work by Gorobets et al.^15^ introduced two types of errors (modifying the number of MU of ‐10% to +10% relative to the original plan or by shifting the isocenter position by 1–5 mm) and compared the measured dose without errors and compared the GPR with those without errors. They found that for an isocenter shift of 3 mm to be detectable the gamma criteria should be at least 2%/2 mm. For MU error detection the GPR results using the 3%/3 mm and 2%/2 mm gamma criteria, the analysis demonstrates that the system is largely insensitive to small dose errors but shows statistically significant sensitivity to larger dose deviations—especially overdoses and in larger targets—due to the normalization of dose differences to the highest dose points when using a global gamma criteria.

Overall, the present results support the clinical feasibility of implementing the OCTAVIUS 4D MR system combined with the OD 1500 MR array for rotational three‐dimensional SBRT patient‐specific QA on a 1.5 T MR‐LINAC under clinically relevant delivery conditions.

## 5 CONCLUSION

This study demonstrates the successful clinical implementation of the MR‐conditional OCTAVIUS 4D rotational phantom combined with the 1500 MR detector array for patient‐specific QA on a 1.5 T MR‐LINAC. Across 40 SBRT treatment plans, the system provided consistent three‐dimensional dose reconstruction with high GPR under clinically relevant delivery conditions. Angular positioning measurements demonstrated accurate and reproducible phantom rotation, while the agreement observed between reconstructed and TPS‐calculated dose distributions supported the effective synchronization of the system during clinical step‐and‐shoot deliveries. In addition to confirming the dosimetric feasibility of MR‐conditional detector arrays, this work highlights the practical integration of the motorized OCTAVIUS 4D system into a clinical MR‐guided radiotherapy workflow.

## AUTHOR CONTRIBUTIONS

CC drafted the manuscript. CC, MB, CM, RS performed the measurement, CC performed the data analysis. CC, MB, CM, RS, RB, IT critically revised the manuscript. All authors read and approved the final manuscript.

## CONFLICT OF INTEREST STATEMENT

The authors have nothing to declare

## ETHICAL STATEMENT

This study involved retrospective analysis of patient‐specific quality assurance data acquired as part of routine clinical practice. No patient‐identifiable information was used. According to institutional policy, formal ethics committee approval was not required.

## Data Availability

The data supporting the findings of this study are available from the corresponding author upon reasonable request.
